# GPR39 deorphanization: The long and winding road to eicosanoids and a crosstalk between GPR39 and hedgehog signaling in angiogenesis

**DOI:** 10.1073/pnas.2308227120

**Published:** 2023-07-03

**Authors:** Urszula Doboszewska, Wolfgang Maret, Piotr Wlaź

**Affiliations:** ^a^Department of Pharmacobiology, Jagiellonian University Medical College, Kraków PL 30-688, Poland; ^b^Department of Nutritional Sciences, School of Life Course and Population Sciences, Faculty of Life Sciences and Medicine, King's College London, London SE1 9NH, United Kingdom; ^c^Department of Animal Physiology and Pharmacology, Institute of Biological Sciences, Maria Curie-Skłodowska University, Lublin PL 20-033, Poland

G-protein-coupled receptors (GPCRs) transduce signals via heterotrimeric G-proteins/β-arrestin and are the most common targets for currently approved drugs. Well-defined ligands and functions have been established for some, but many remain orphan receptors.

**Fig. 1.**
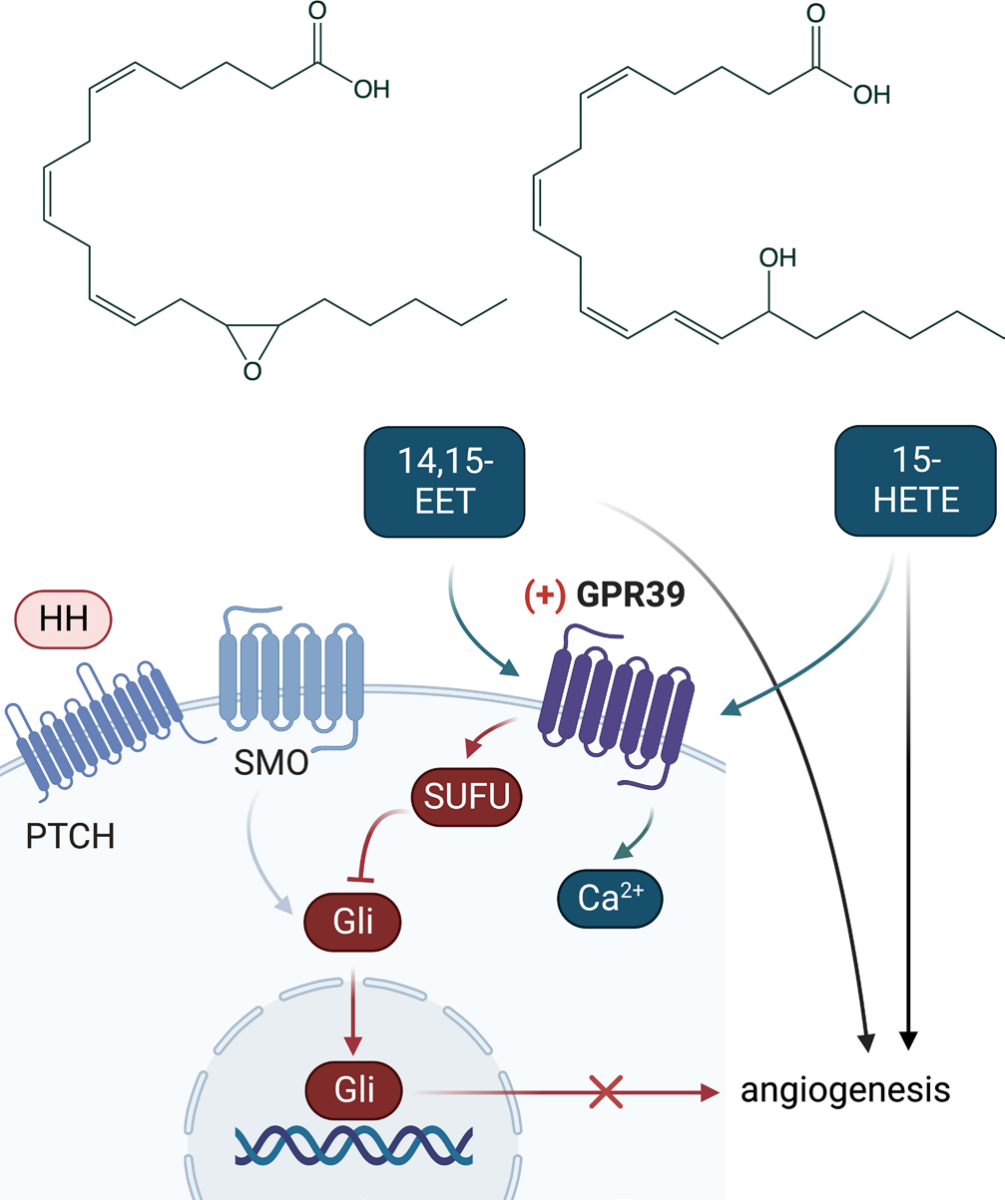
Hedgehog (HH) signaling pathway regulates angiogenesis. The HH protein signals to the transmembrane protein Patched (PTCH). The activation of PTCH by HH relieves PTCH inhibition of the GPCR-like protein Smoothened (SMO), which then signals downstream to the glioma-associated oncogene transcription factor (Gli). In PNAS, Meda Venkata et al. (1) demonstrated a role for the G-protein-coupled receptor 39 (GPR39) in the effects of HH on angiogenesis. They showed that activation of GPR39 inhibits angiogenesis by inhibiting HH signaling. They also demonstrated that inhibition of GPR39 increases HH signaling through activating Gli via decreased binding of GPR39 to the endogenous inhibitor of the pathway, the suppressor of fused (SUFU). Last year, Alkayed et al. (2) showed that GPR39 is a receptor for two eicosanoids belonging to epoxyeicosatrienoic acids (EETs) and hydroxyeicosatetraenoic acids (HETEs): 14,15-EET and 15-HETE, respectively. They demonstrated that 15-HETE stimulates GPR39 signaling by increasing the second-messenger Ca^2+^, while 14,15-EET inhibits this action. The crosstalk of these pathways provides ample new opportunities to explore the role of GPR39 and develop new therapeutic drugs.

In PNAS, Meda Venkata et al. (1) describe a novel role for an orphan GPCR, namely GPR39. They showed that the GPR39 knockout (KO) mice displayed a faster pace of revascularization from hind limb ischemia and a lower incidence of tissue necrosis than those of their WT littermates, thus indicating that GPR39 inhibition would be a way of protection against peripheral artery disease. They demonstrated that the underlying mechanism involves stimulation of angiogenesis by the hedgehog (HH) signaling pathway (Fig. 1). The authors state that it was beyond the scope of their investigation to search for endogenous ligand(s) mediating the effects. New work on eicosanoids as ligands provides a new context for these observations (2).

Eicosanoids such as epoxyeicosatrienoic acids (EETs) and hydroxyeicosatetraenoic acids (HETEs) are signaling molecules that are involved in a variety of physiological or pathological processes in the vasculature, such as angiogenesis, ischemia, or blood pressure control. Increasing evidence suggests that their actions are mediated via specific membrane receptors, at least in part via GPCRs (3). Congruently, last year, GPR39 was suggested to be a receptor for 14,15-epoxyeicosatrienoic acid (14,15-EET) as an antagonist and 15-HETE as an agonist, the actions of which are allosterically modulated by Zn^2+^ (2).

This observation adds another major dimension to the work published in PNAS (1). Both EETs and HETEs [such as 15(S)-HETE] were demonstrated to enhance angiogenesis (3, 4). 15(S)-HETE was already known to stimulate angiogenesis in response to hind limb ischemia (4). The effects of GPR39 KO in response to hind limb ischemia are thus convergent to the effects of 15(S)-HETE, both stimulating angiogenesis. On the contrary, the effects of GPR39 KO or GPR39 antagonists on blood pressure described in the patent literature, i.e., decreasing blood pressure (5), are opposite to the effects of 15-HETE, which induces pulmonary hypertension (6). Therefore, future studies should be directed toward establishing whether the effects of 15-HETE and 14,15-EET on GPR39 are restricted to the effects on the vasculature or whether eicosanoid ligands can explain the reported effects of GPR39 in many other tissues.

Previously, obestatin (7) or Zn^2+^ (8) was proposed as an endogenous agonist for GPR39. The findings on obestatin, however, were refuted. The existence of a GPCR activated by Zn^2+^ (Zn^2+^-sensing receptor) was proposed in PNAS in 2001 (9) and later identified as GPR39. Given the EC_50_ (8) or *K*_d_ (9) values for Zn^2+^, it is debatable whether the physiological concentrations of Zn^2+^ are sufficient to activate the receptor (10). Instead, Zn^2+^ may be an allosteric effector as in the case of several other GPCRs. The new investigation on eicosanoids (2) supports the latter possibility. Resolving the issue of the endogenous ligand(s) of GPR39 would not only deorphanize this receptor but also contribute significantly to progress in basic sciences and validation of GPR39 as a drug target.
